# Changes of Peripapillary Capillary Density in Patients with Vogt–Koyanagi–Harada Disease Evaluated by Optical Coherence Tomography Angiography

**DOI:** 10.1155/2023/1271070

**Published:** 2023-04-17

**Authors:** Zhijian Jiang, Huiying Ji, Nan Zhang, Liang Huang, Min Zhou, Jianhong Dong

**Affiliations:** ^1^Department of Ophthalmology, Shanghai Xuhui Central Hospital, Shanghai 200031, China; ^2^Department of Laboratory, Shanghai Xuhui Central Hospital, Shanghai 200031, China; ^3^Department of Ophthalmology, Eye Ear Nose and Throat Hospital of Fudan University, Shanghai 200031, China

## Abstract

**Objective:**

To analyze the longitudinal changes in peripapillary capillary density in patients with acute VKH with or without optic disc swelling by optical coherence tomography angiography (OCTA).

**Methods:**

Retrospective case series. 44 patients (88 eyes) were enrolled and were divided into two groups according to presence/absence of optic disc swelling before treatment. Peripapillary capillary images were obtained by OCTA before and after 6 months of corticosteroid treatment and used to determine the radial peripapillary capillary (RPC), retinal plexus, and choriocapillaris vessel perfusion densities.

**Results:**

Optic disc swelling was present in 12 patients (24 eyes) and absent in 32 patients (64 eyes). The sex distribution, age, intraocular pressure, and best-corrected visual acuity before and after treatment were not significantly different between the two groups (all *P* > 0.05). Compared to those in nonoptic disc swelling group, the percentages of decreased vessel perfusion densities after treatment in the supranasal (RPC, 100.00% vs. 75.00%), infranasal (RPC, 100.00% vs. 56.25%), infratemporal (RPC, 66.67% vs. 37.50%), and infranasal quadrants (retinal plexus, 83.33% vs. 56.25%) were significantly more in optic disc swelling group. The choriocapillaris vessel perfusion density increased after treatment in both groups.

**Conclusions:**

Decreases in vessel perfusion densities of the RPC and retinal plexus after treatment in VKH patients with optic disc swelling were more common than in those without optic disc swelling. The choriocapillaris vessel perfusion density increased after treatment, regardless of the presence/absence of optic disc swelling.

## 1. Background

Vogt–Koyanagi–Harada (VKH) disease is an autoimmune disease that affects multiple systems, including eyes, skin, inner ear, and meninges. It is common in China and its etiology is unknown [1]. Although the most common ocular feature of VKH disease is serous retinal detachment [2], 14.6%–27.6% of patients in prior reports presented with more significant optic disc swelling without serous retinal detachment [3–5]. In some patients, optic disc swelling may eventually lead to visual field defects [5].

Multimodal imaging technologies are widely used for the diagnosis of VKH disease and to monitor the effectiveness of its treatment. These technologies include fundus photography, autofluorescence, fluorescence angiography (FA), indocyanine green angiography (ICGA), and optical coherence tomography (OCT). FA and ICGA have irreplaceable advantages such as the display of vascular leakage but are invasive examinations with a risk of allergic reaction to the dye. Autofluorescence can reveal the function of the retinal pigment epithelium, and OCT can depict (at high resolution) any structural abnormalities caused by serous retinal detachment and optic disc swelling [6]. In recent years, optical coherence tomography angiography (OCTA), a noninvasive technology, has been used to observe the microcirculation and blood flow through the retina and choroid. Several studies have revealed abnormal macular vessel densities of the retina and choroid in patients with VKH disease [7–10], but no studies have reported the changes in peripapillary capillary density. Therefore, we performed a study of patients with acute VKH disease and compared peripapillary capillary densities before and after treatment.

## 2. Methods

### 2.1. Participants

We performed a retrospective study of patients, all Chinese Han, with acute VKH disease who were hospitalized at Shanghai Xuhui Central Hospital (Shanghai, China) between June 2020 and September 2021. This study adhered to the tenets of the Declaration of Helsinki and was approved by the Ethics Committee of Shanghai Xuhui Central Hospital. All of the patients satisfied the diagnostic criteria for acute VKH disease (as described in section Diagnostic criteria for acute VKH disease). Patients with a history of other systemic immune diseases, developmental abnormalities, genetic diseases, or other ocular diseases such as corneal diseases, glaucoma, or degeneration were excluded. Patients previously treated with a corticosteroid or immunosuppressant and patients who could not cooperate with the examinations were also excluded. At the time of treatment, patients provided consent for future use of their data for research purposes.

All patients underwent systematic eye examinations, including OCTA. The patients were divided into two groups according to the presence or absence of optic disc swelling based on their clinical manifestations and imaging characteristics before treatment. Optic disc swelling was defined as follows: optic disc swelling was clearly visible on fundus examination; if it was not obvious on fundus examination, OCT revealed optic disc swelling and mild serous retinal detachment. Patients who did not meet these criteria were included in the nonoptic disc swelling group.

The best-corrected visual acuity (BCVA), interval between the onset of ocular symptoms and initiation of treatment, and the intraocular pressure before and after treatment were recorded in all patients. BCVA was converted to logarithms of the minimum angle of resolution (logMAR) for analysis.

### 2.2. Diagnostic Criteria for Acute VKH Disease

The diagnostic criteria for acute VKH were as follows [11]: (A) no history of penetrating ocular trauma or intraocular surgery preceding the initial onset of uveitis. (B) Bilateral ocular involvement (time interval between the 2 eyes should be ≤2 weeks). (C) No history nor clinical evidence of ocular tuberculosis, syphilis, or ocular toxoplasmosis; no underlying systemic rheumatic disease that could explain the form of uveitis, and no history or clinical evidence to suggest the possibility of a specific entity (e.g., intraocular tumors, toxic uveitis, Fuchs syndrome, or Posner–Schlossman syndrome). (D1) Signs of diffuse choroiditis and exudative retinal detachment. (D2) Serous retinal detachment detected by OCT or B-scan ultrasonography. (D3) Choroidal thickening on enhanced depth imaging OCT. (D4) Early punctate staining and late subretinal dye pooling on FA. (D5) Hyperfluorescence of the optic disc on FA. Only patients who satisfied criteria A–C plus either D1, D2, D3, or D4 were considered to have acute VKH disease.

### 2.3. Therapeutics of Acute VKH Disease

Following diagnosis of acute VKH, patients started intravenous methylprednisolone at a dose of 1.0 or 0.5 g/d for 3 days according to the severity of the disease. The dose was halved every 3 days until 0.25 g/d, which was continued for 3 days. This was followed by oral corticosteroid therapy with a tapered dose regimen. Corticosteroid therapy was planned to continue for more than 6 months after disease onset.

### 2.4. OCTA Examination of the Peripapillary Area

OCTA was performed upon admission to the hospital and after 6 months of treatment for VKH. All patients were scanned in the AngioPlex ONH mode to obtain blood flow images with an area of 4.5 × 4.5 mm around the optic disc using an OCTA scanner (Cirrus HD-OCT 5000, Zeiss, CA., USA) with software version 9.6. The software provides automatic segmentation to analyze *en face* images and depict the radial peripapillary capillary (RPC; derived from the internal limiting membrane [ILM] to the lower boundary of retinal nerve fiber layer [RNFL]), the total retinal plexus (derived from the ILM to the outer boundary of outer plexiform layer), and choriocapillaris (derived from a 10 *μ*m slab located 31–40 *μ*m below the retinal pigment epithelium). Any notable segmentation error was corrected manually. Projection artifacts caused by overlying retinal vessels were removed by the built-in software. All images were obtained by the same experienced doctor (Zhijian Jiang). The qualities of the images were graded automatically by the device on a 10-point scale, where Q1 (1/10) is the worst and Q10 (10/10) is the best. Only images graded Q6 or above were included in this study. Images with substantial motion artifact were excluded. The *en face* OCTA images, centered on the optic disc, were divided into the supranasal, infranasal, supratemporal, and infratemporal quadrant.

**Figure 1:**
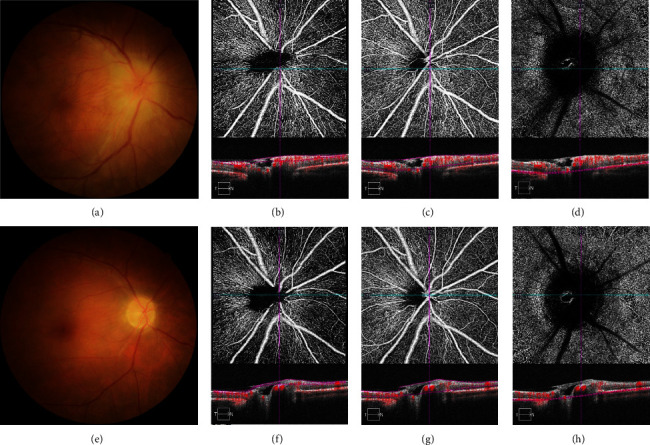
Representative fundus photographs and OCTA images of the right eye of a 50-year-old female with acute VKH who presented with significant optic disc swelling. Images were taken before (a–d) and after (e–h) treatment for 6 months. (a) Fundus photograph before treatment revealing radial folds between the macular and optic disc and evidence of macular swelling. (e) Fundus photograph taken after treatment revealing a gray optic disc with a clear boundary and a slightly thinner artery. (b, f) The RPC vessel perfusion density decreased after treatment in all four quadrants. (c, g) The retinal plexus vessel perfusion density decreased after treatment in the infranasal, supratemporal, and infratemporal quadrants, but increased in the supranasal quadrant. (d, h) The choriocapillaris vessel perfusion density increased after treatment in all four quadrants.

**Figure 2:**
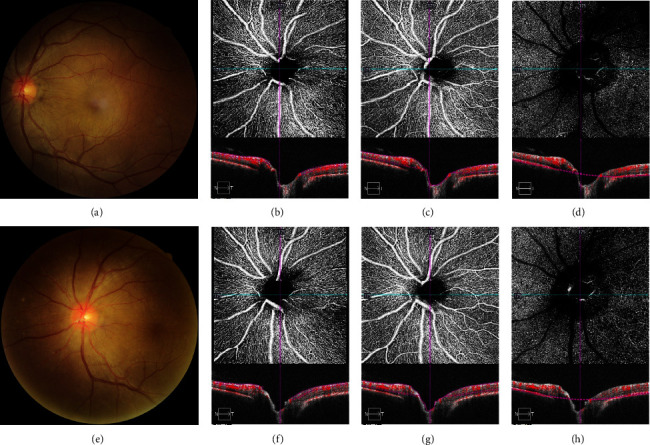
Representative fundus photographs and OCTA images of the left eye of a 46-year-old female with acute VKH disease who presented without optic disc swelling. Images were taken before (a–d) and after (e–h) treatment for 6 months. (a) Fundus photograph revealing serous retinal detachment and macular swelling. (e) Normal fundus photograph after treatment. (b, f) The RPC vessel perfusion density increased after treatment in the supranasal, infranasal, and supratemporal quadrants, but not the infratemporal quadrant. (c, g) The retinal plexus vessel perfusion density increased after treatment in all quadrants. (d, h) The choriocapillaris vessel perfusion densities increased after treatment in all four quadrants.

According to the images, two independent and experienced examiners (Huiying Ji and Liang Huang) qualitatively evaluated the changes in peripapillary capillary densities in each quadrant before and after treatment. First, a pair of images with the same layer of peripapillary capillary before and after treatment were magnified by 5 times. Then, each quadrant was divided into 100 squares on average. The changes of vessel perfusion density in each small square were compared, and the number of squares with increased and decreased vessel perfusion density were recorded, respectively. When the number of squares with increased vessel perfusion density was more than the number of squares with decreased vessel perfusion density, it was determined that the vessel perfusion density in this quadrant increased. And vice versa, the vessel perfusion density in this quadrant decreased. Divided the number of eyes with decreased/increased vessel density by the sample size of the group to calculate the percentage of decreased/increased vessel density of the group in each quadrant. Representative fundus photographs and OCTA images of eyes with VKH disease with or without optic disc swelling are shown in Figures 1 and 2, respectively.

### 2.5. Statistical Analysis

SPSS version 15.0 (SPSS Inc., Chicago, IL, USA) was used for statistical analyses. Normally distributed data are presented as the mean ± standard deviation and the non-normal distribution data is present as the median and range/interquartile range. The Kolmogorov–Smirnov test was used to test for normal distributions. The independent samples *t* test was used to compare normally distributed variables, and the Mann–Whitney *U* test was used to compare non-normally distributed variables between patients with and without optic disc swelling. The Wilcoxon test was used to compare non-normally distributed variables from before and after treatment in each group. The *χ*^2^ test was used to test categorical variables between groups and/or before and after treatment. While if the minimum expected count less than 5, Fisher's exact test was used. In all analyses, *P* < 0.05 was considered statistically significant.

## 3. Results

### 3.1. General Clinical Characteristics

A total of 44 patients (88 eyes) were enrolled, of which 23 were male (46 eyes, 52.27%). The mean age was 45.66 ± 13.50 years (range 17–66 years) and the interval between the onset of ocular symptoms and initiation of treatment was 16.98 ± 15.08 days. Among them, 12 cases (24 eyes, 27.27%) were in the optic disc swelling group and 32 cases (64 eyes) were in nonoptic disc swelling group. The demographic and clinical characteristics of these two groups are shown in Table 1. There were no significant differences between the two groups in terms of the sex distribution (*P* > 0.05), age (*P* > 0.05), or intraocular pressure (*P* > 0.05). However, the interval between the onset of ocular symptoms and initiation of treatment was significantly longer in patients with optic disc swelling (*P* < 0.01). Although there were no differences in BCVA (logMAR) between the two groups before or after treatment (both *P* > 0.05), BCVA improved significantly after treatment in both groups (both *P* < 0.001).

### 3.2. Peripapillary Capillary Densities Determined by OCTA

The changes in peripapillary capillary density after 6 months corticosteroid treatment in patients with and without optic disc swelling are shown in Table 2. The percentages of decreased RPC vessel density of the supranasal, infranasal, supratentoral, and infratemporal quadrants were 100.00%, 100.00%, 66.67%, and 66.67%, respectively, in patients with optic disc swelling. The corresponding values in patients without optic disc swelling were 75.00%, 56.25%, 50.00%, and 37.50%, respectively. There were significant differences in the percentages of decreased vessel perfusion densities of the supranasal (*P* < 0.01), infranasal (*P* < 0.001), and infratemporal (*P* < 0.05) quadrants between the two groups.

The percentages of increased RPC vessel density of the supranasal, infranasal, supratentoral, and infratemporal quadrants were 0.00%, 0.00%, 29.17%, and 29.17%, respectively, in patients with optic disc swelling. The corresponding values in patients without optic disc swelling were 20.31%, 37.5%, 39.06%, and 59.38%, respectively. There were significant differences in the percentages of decreased vessel perfusion densities of the supranasal (*P* < 0.05), infranasal (*P* < 0.001), and infratemporal (*P* < 0.05) quadrants between the two groups.

The percentages of decreased retinal plexus vessel density of the supranasal, infranasal, supratentoral, and infratemporal quadrants were 83.33%, 83.33%, 66.67%, and 66.67%, respectively, in patients with optic disc swelling. The corresponding values in patients without optic disc swelling were 75.00%, 56.25%, 43.75%, and 50.00%, respectively. The difference of the percentages of decreased retinal plexus vessel density in infranasal quadrant were significant between the two groups (*P* < 0.05).

The percentages of increased retinal plexus vessel density of the supranasal, infranasal, supratentoral, and infratemporal quadrants were 12.50%, 12.50%, 29.17%, and 25.00%, respectively, in patients with optic disc swelling. The corresponding values in patients without optic disc swelling were 20.31%, 37.50%, 50.00%, and 46.88%, respectively. The difference of the percentages of increased retinal plexus vessel density in infranasal quadrant were significant between the two groups (*P* < 0.05).

The percentages of increased choriocapillaris vessel density increased in each quadrant after treatment were 100%, whether in optic disc swelling group or in nonoptic disc swelling group.

## 4. Discussion

This study is the first to evaluate the changes in peripapillary capillary perfusion, using OCTA, in patients with VKH disease. We found that the vessel perfusion densities of the RPC and retinal plexus decreased after treatment in most VKH patients with optic disc swelling. By comparison, the percentages of decreased peripapillary capillary density in the infranasal, supratemporal, and infratemporal quadrant were lower after treatment in VKH patients without disc swelling. The choriocapillaris vessel density increased after treatment regardless of the presence or absence of optic disc swelling.

VKH disease is an autoimmune disease that primarily targets melanocytes and is characterized by bilateral granulomatous panuveitis with neurosensory symptoms such as impaired hearing, tinnitus, and vitiligo [1, 12]. Owing to the abundance of melanocytes in choroidal tissue, T cells mediate an autoimmune response of melanocytes that leads to choroidal matrix inflammation and a series of ocular manifestations due to the involvement of the retinal pigment epithelium and retina. Serous retinal detachment is the most common ocular manifestation of VKH in the acute stage [2], of which the positive and negative predicting diagnosis of VKH were 100% and 89.2%, respectively [13]. Optic disc swelling is the second most common ocular manifestation. In prior studies, 14.6%–27.6% of patients with acute VKH disease showed significant optic disc swelling with or without slight serous retinal detachment [3–5].

In a study of patients with acute VKH, Okunuki reported that compared with patients with significant serous retinal detachment, patients with optic disc swelling are older, more commonly female, and more likely to suffer chronic recurrence of VKH disease [4]. In another study of patients with VKH, Nakao reported that those with optic disc swelling were older compared with patients with serous retinal detachment, but they found no difference in the sex distribution [5]. In our study, there were no significant differences in age or sex composition between patients with or without optic disc swelling, which is consistent with the results of our previous study [3]. Thus, possible discrepancies in earlier studies may be due to population or racial differences or sampling errors. In this study, patients with optic disc swelling had a longer interval between the onset of ocular symptom onset and the initiation of treatment but similar visual acuity compared with patients without optic disc swelling. A possible reason is that most cases of serous retinal detachment involve the macular region, and the decline in visual acuity is more obvious with the same onset time, which prompts patients to seek medical treatment as soon as possible. By comparison, effects on visual acuity are minimal in the initial stage of VKH disease in patients with optic disc swelling, and the patient may delay seeking treatment. Another reason may be that the diagnosis of VKH is delayed in patients whose main manifestation is optic disc swelling. Such patients may have little or no exudative retinal detachment and could be misdiagnosed as other diseases, such as optic neuritis or intracranial hypertension, prolonging the time to a correct diagnosis. Therefore, the possibility of VKH should be strongly considered in patients with VKH presenting with optic disc swelling as the main manifestation. Despite the long interval between ocular symptom onset and the initiation of treatment, the visual acuity of VKH patients with optic disc swelling recovered well after corticosteroid therapy, similar to that of VKH patients with serous retinal detachment.

In the acute stage of VKH, the thickened choroid, caused by inflammatory cell infiltration and necrotizing granuloma, may compress and damage the choriocapillaris, resulting in abnormal blood flow [14]. Delayed filling or hypoperfusion of the choriocapillaris can be detected by ICGA in acute VKH disease [15, 16]. However, ICGA has some limitations including its invasiveness and the risk of dye-related complications. OCTA can depict blood flow perfusion by detecting the motion of red blood cells and provide images of the retinal and choroidal microvascular system in a noninvasive manner [8]. Some studies of OCTA of the choriocapillaris have shown multiple flow void spots and subthreshold perfusion, which correlate with hypoperfusion lesions detected by ICGA [9, 10, 17, 18]. In addition, OCTA can be used for layered quantitative and qualitative analyses of the retina and choroid that are not possible with ICGA or FFA. Ye reported that the densities of the superficial and deep retinal vascular plexuses decreased in patients with acute VKH disease compared with healthy controls [9]. In convalescent VKH patients with sunset glow fundus, Fan found that the vessel densities of superficial capillaris plexus and the deep capillaris plexus decreased significantly compared with those without sunset glow fundus [19]. Liang reported that in the acute and chronic recurrent stages of VKH disease, the vessel densities of the deep capillary plexus decreased significantly and correlated with history of anterior recurrence [20]. This suggests that the vessel densities of the deep capillary plexus may be a sensitive indicator of the inflammatory state in patients with VKH disease. All of the studies described above focused on the microcirculation and perfusion in the macular region, and none examined peripapillary capillary perfusion in patients with VKH disease.

The present study evaluated the changes in choriocapillaris vessel densities around the disc following treatment in patients with acute VKH. We found that the choriocapillaris vessel density increased after treatment in all of the patients with acute VKH, regardless of the presence or absence of optic disc swelling. The results are consistent with the changes of choriocapillaris vessel density in the macular reported in prior studies [9, 10, 17]. This shows that although there are differences in clinical features between patients with optic disc swelling and serous retinal detachment, choroiditis is the predominant manifestation in VKH disease. Inflammation could diffuse damage to the choriocapillaris, decreasing the choriocapillaris vessel densities of the macular and peripapillary. Following treatment of VKH, the damaged capillaries gradually recover as the inflammatory state subsides.

We also showed that the vessel perfusion densities of the RPC and retinal plexus decreased in most VKH patients with optic disc swelling. While in more VKH patients without optic disc swelling, RPC and retinal plexus vascular densities increased after treatment. This suggests that the pathogenesis of VKH differs between patients with and without optic disc swelling. The RPCs located in the RNFL originate from arterioles in the ganglion cell layer. In healthy subjects, the average RPC plexus density was positively correlated with the average thickness of the RNFL [21, 22], indicating that the RPC plexus supplies the RNFL around the optic nerve head [23]. Prior studies have also reported decreases in RPC density in patients with glaucoma, Fuch's syndrome, and nonarteritic anterior ischemic optic neuropathy, all of which are characterized by visual field defects [24–26]. In some case reports, VKH disease with optic disc swelling was accompanied by anterior ischemic optic neuropathy (AION), resulting in a reduced RNFL thickness and visual field defects, but AION was not found in patients with VKH disease without optic disc swelling [27, 28]. Although optic disc swelling is a risk factor for AION, there were no cases of AION in any of the patients in this study, and AION is a rare complication of VKH. Compared with those 6 months after treatment, the relative increases in the RPC and retinal plexus vessel perfusion densities in acute VKH patients with optic disc swelling might be a compensatory response to local inflammation-induced ischemia aimed at increasing blood flow through a region with a reduced vessel density. However, more severe inflammatory obstruction or other reasons might impede this compensatory process in patients without optic disc swelling, and it is therefore characterized by reduced vessel perfusion densities. However, the vessel density of the peripapillary capillaries tended to normalize after treatment in patients with or without optic disc swelling.

The following limitations of our study should be noted. The retrospective study design and small sample size may introduce some bias. In addition, owing to limitations of the equipment, the Cirrus HD-OCT 5000 with software version 9.6 can only quantify macular capillary perfusion and cannot quantitatively analyze the vessel perfusion densities of the RPC plexus or other layers around the optic disc. Despite these limitations, the results of this longitudinal qualitative study have some research value.

## 5. Conclusion

Optic disc swelling is an important clinical manifestation of VKH disease, which should be considered in patients presenting with optic disc swelling. The changes of peripapillary capillary density in VKH patients after treatment is different with the presence or absence of optic disc swelling. Decreases in the vessel perfusion densities of the RPC and retinal plexus after treatment in patients with optic disc swelling are more common than in those without optic disc swelling. VKH patients without optic disc swelling have a higher proportion of increased vessel perfusion densities of RPC and retinal plexus after treatment. The choriocapillaris vessel perfusion density was decreased at the initial stage in patients with acute VKH disease and increased gradually after treatment, regardless of the presence or absence of optic disc swelling.

## Figures and Tables

**Table 1:** Demographic and clinical data of patients with VKH disease with or without optic disc swelling.

	Optic disc swelling group	Nonoptic disc swelling group	*P* value
*N*	12	32	—
Sex (M/F)	6/6	17/15	0.853^a^
Age ± SD, year	50.50 ± 9.64	43.84 ± 14.40	0.088^b^
Interval between ocular symptom onset and initiation of treatment, median (interquartile range), days	29.00 (10.50, 31.50)	7.00 (5.25, 19.00)	0.003^c^
Pretreatment IOP ± SD, mmHg	15.00 ± 1.89	14.68 ± 2.45	0.576^b^
Post-treatment IOP ± SD, mmHg	15.27 ± 1.47	14.83 ± 2.22	0.367^b^
Pretreatment BCVA (logMAR), median (interquartile range)	0.60 (0.33, 1.05)	0.65 (0.40, 0.90)	0.814^c^
Post-treatment BCVA (logMAR), median (interquartile range)	0.10 (0.00, 0.28)	0.00 (0.00, 0.10)	0.053^c^

SD standard deviation, IOP intraocular pressure, BCVA best-corrected visual acuity. ^a^*χ*^2^ test. ^b^*t* test. ^c^Mann–Whitney *U* test.

**Table 2:** Changes of peripapillary capillary density after treatment in VKH patients with or without optic disc swelling.

		Optic disc swelling group (eye number of decreased capillary density/eye number of increased capillary density)	Nonoptic disc swelling group [eye number of decreased capillary density/eye number of increased capillary density]	*P* value (the percentage of decreased vessel density)	*P* value (the percentage of increased vessel density)
RPC	Supranasal	24/0	48/13	0.005^a^	0.016^a^
	Infranasal	24/0	36/24	<0.001^b^	<0.001^b^
	Supratemporal	16/7	32/25	0.162^b^	0.390^b^
	Infratemporal	16/7	24/38	0.014^b^	0.012^b^

Retinal plexus	Supranasal	20/3	48/13	0.406^b^	0.541^a^
	Infranasal	20/3	36/24	0.019^b^	0.024^b^
	Supratemporal	16/7	28/32	0.056^b^	0.080^b^
	Infratemporal	16/6	32/30	0.162^b^	0.063^b^

^a^Fisher's exact test. ^b^*χ*^2^ test.

## Data Availability

All datasets used and/or analysed in the current study are available from the corresponding author upon reasonable request.
